# Supplementary material for: Impact of porcine cytomegalovirus on long-term orthotopic cardiac xenotransplant survival
Source: Sci Rep. 2020 Oct 16;10:17531. doi: 10.1038/s41598-020-73150-9 (PMC7568528; doi:10.1038/s41598-020-73150-9)
Supplement: Supplementary file 1 — Supplementary Figure 1 [file 41598_2020_73150_MOESM1_ESM.pdf]

Supplementary Figure 1

Impact of porcine cytomegalovirus on long-term orthotopic cardiac xenotransplant survival

Joachim Denner, Matthias Längin, Bruno Reichart, Luise Krüger, Uwe Fiebig, Maren Mokelke, Julia Radan, Tanja Mayr, Anastasia Milusev, Fabian Luther, Nicoletta Sorvillo, Robert Rieben, Paolo Brenner, Christoph Walz, Eckhard Wolf, Berit Roshani, Christiane Stahl-Hennig, and Jan-Michael Abicht.

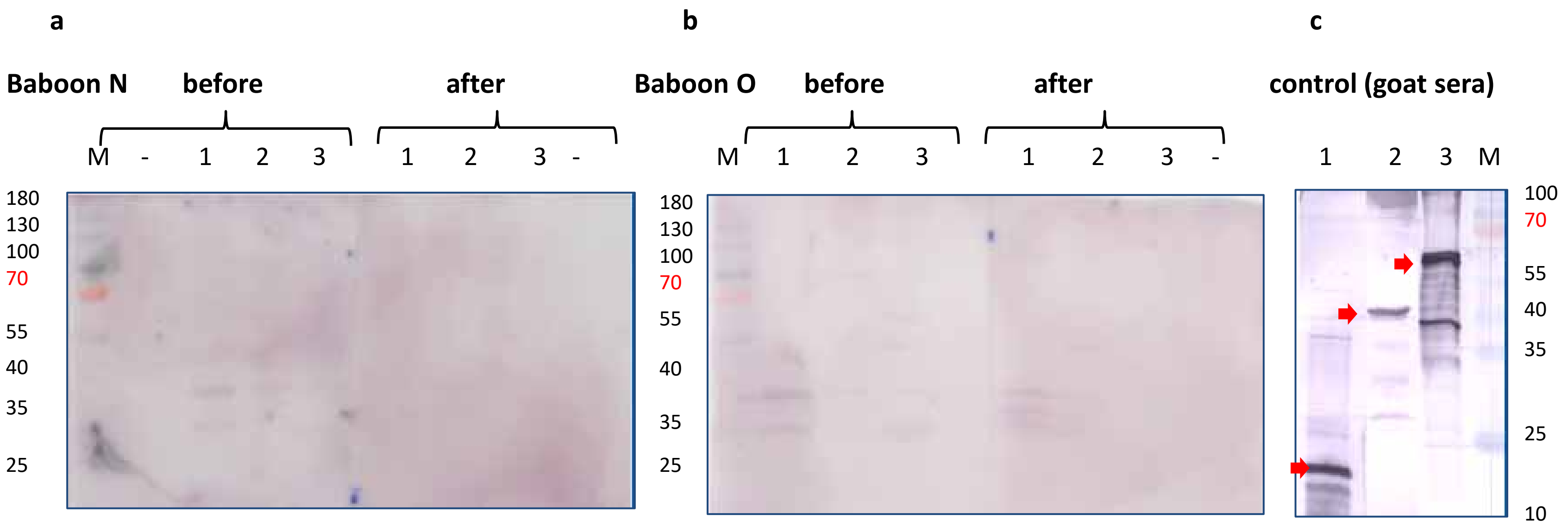

Supplementary Figure 1 Western blot analysis of sera from baboon N and O before transplantation (before) and at the end of the experiment (after). **a** serum from baboon N, **b** serum from baboon O, **c** control sera obtained in goats against recombinant PERV antigens. 1, recombinant p15E, 2, recombinant p27Gag, 3, recombinant gp70. The red arrows indicate the recombinant proteins. The blots were overexposed to increase sensitivity and show unspecific bands.
